# Culture of Primary Neurons from Dissociated and Cryopreserved Mouse Trigeminal Ganglion

**DOI:** 10.1089/ten.tec.2023.0054

**Published:** 2023-08-08

**Authors:** Molly Tzu-Yu Lin, Isabelle Xin Yu Lee, Wei-Li Chen, Mei-Yun Chen, Jodhbir S. Mehta, Gary H. F. Yam, Gary S. L. Peh, Yu-Chi Liu

**Affiliations:** ^1^ Tissue Engineering and Cell Therapy Group, Singapore Eye Research Institute, Singapore, Singapore.; ^2^ Department of Ophthalmology, National Taiwan University Hospital, Taipei, Taiwan.; ^3^ Advanced Ocular Surface and Corneal Nerve Research Center, National Taiwan University, Taipei, Taiwan.; ^4^ Department of Ophthalmology, College of Medicine, National Taiwan University, Taipei, Taiwan.; ^5^ Corneal and External Eye Disease Department, Singapore National Eye Centre, Singapore, Singapore.; ^6^ Ophthalmology and Visual Sciences Academic Clinical Program, Duke-NUS Medical School, Singapore, Singapore.; ^7^ Department of Ophthalmology, University of Pittsburgh, Pittsburgh, Pennsylvania, USA.

**Keywords:** corneal nerve, trigeminal ganglion, primary neuronal culture, cryopreservation

## Abstract

Corneal nerves originate from the ophthalmic branch of the trigeminal nerve, which enters the cornea at the limbus radially from all directions toward the central cornea. The cell bodies of the sensory neurons of trigeminal nerve are located in the trigeminal ganglion (TG), while the axons are extended into the three divisions, including ophthalmic branch that supplies corneal nerves. Study of primary neuronal cultures established from the TG fibers can therefore provide a knowledge basis for corneal nerve biology and potentially be developed as an *in vitro* platform for drug testing. However, setting up primary neuron cultures from animal TG has been dubious with inconsistency among laboratories due to a lack of efficient isolation protocol, resulting in low yield and heterogenous cultures. In this study, we used a combined enzymatic digestion with collagenase and TrypLE to dissociate mouse TG while preserving nerve cell viability. A subsequent discontinuous Percoll density gradient followed by mitotic inhibitor treatment effectively diminished the contamination of non-neuronal cells. Using this method, we reproducibly generated high yield and homogenous primary TG neuron cultures. Similar efficiency of nerve cell isolation and culture was further obtained for TG tissue cryopreserved for short (1 week) and long duration (3 months), compared to freshly isolated tissues. In conclusion, this optimized protocol shows a promising potential to standardize TG nerve culture and generate a high-quality corneal nerve model for drug testing and neurotoxicity studies.

**Impact statement:**

Isolation of neurons from trigeminal ganglion (TG) has been used for deeper insights on corneal nerve biology. However, the lack of standardized isolation protocols may be the attributes of the heterogenous culture with inconsistent results among laboratories. In this study, we investigated an optimized protocol for TG neuron culture with good efficiency. Furthermore, compared with freshly prepared TG culture, the culture prepared with cryopreserved TG tissues or neurons showed similar morphology with preserved neuronal marker expression. This study highlights the promising potential of using optimized protocol as a standardized isolation method as *in vitro* corneal nerve model for corneal nerve studies.

## Introduction

The cornea is the most densely innervated tissue in the body, with an abundance of sensory nerve supply (∼7000 sensory neurons per mm^2^).^1^ The corneal nerves originate from the ophthalmic branch (V1) of the TG, and the sensory nerve fibers enter at the corneal anterior and middle stroma through the limbal area, in a radially inward manner toward the central cornea. The stromal nerve fibers then turn perpendicularly and penetrate Bowman's layer before dividing into several smaller nerve branches and progressively innervate upwards to the corneal epithelium with free sensory nerve endings.^1^ Besides the important sensory functions, corneal nerves also play important roles in maintaining the structural and functional integrity of the ocular surface, which include promoting corneal epithelial homeostasis by releasing trophic substances and inducing reflex tear production and blinking by activating brainstem circuits.^2^

Systematic diseases such as diabetes mellitus,^3–5^ as well as infection, inflammation, or surgical intervention, could lead to damage or dysfunction of corneal nerves. Subsequent disruption of the corneal nerves not only results in diminished corneal sensitivity but also compromises the ocular surface integrity and tear film dynamics, resulting in neurotrophic keratopathy and potentially blindness.^6^

The structure, morphology, and function of corneal nerves are influenced by a variety of ophthalmic and systemic conditions.^7^ Developing tests that can assess the status of corneal nerve structure and function with good diagnostic capability is therefore essential to provide prompt intervention. In the clinical setting, several assays are available to assess the structure of corneal nerves and the response to the external stimuli. These include (1) Corneal *in vivo* confocal microscopy (IVCM), which has been widely used since 1990s.^8^ It provides real-time imaging, with high resolution of corneal nerve morphology and distribution profile.^9^ However, the inherent limitation of current IVCM is that it provides a small region of interest (<500 μm^2^).^9^ (2) Corneal sensitivity test as the functional test. This simple test involves touching the cornea gently to initiate a blink response.^10^ Using the Cochet-Bonnet esthesiometer, it gives a rapid and quantitative measurement of corneal sensitivity.^11^ However, the clinical nerve function and nerve morphology may not always correlate.^6,12^ In the *in vitro* culture setting, there is still a lack of assays that can provide insight into the nerve fiber structure and functions, as well as the neuronal innate immune responses in case of viral pathogenesis. The primary culture of corneal neurons thus has an outstanding value for the study of neuronal anatomy, physiology, and functions. Although different neuronal cell lines are available with ease to culture and are proliferative, there are caveats due to their immortalization and mitotic nature that the study results cannot truly reflect the neurons *in vivo*. TG contains neuronal cell bodies of the trigeminal nerves which include the ophthalmic branch that supplies sensory innervation to the cornea.^13^ By virtue of its anatomic and physiological feature it is a suitable source for isolating primary sensory neurons from TG for studies associated to corneal nerves.

Primary neuronal cultures are a valuable resource, which are extensively used as a model to elucidate fundamental aspects of neuronal anatomy, physiology, cellular biology, and neuronal dysfunction in diseased model animals.^14^ The tight control of neuronal and extracellular environment makes it a favorable candidate for physiological and pharmacological studies, of intrinsic electric properties of neurons and sensory transduction.^15^ In addition, the isolated sensory neurons retain the capacity to respond to chemical,^16,17^ thermal,^18^ and mechanical stimuli^19^ in culture and are commonly used to assess neuron-cell interactions and the underlying mechanisms. Despite having enormous potential for vast applications in sensory nerve studies, the primary neuronal culture setup is however time consuming and labor intensive.^14^ High quality of primary neuronal cultures with good consistency is often hard to achieve.^14^ In addition, the lack of standardized isolation protocol might be the main contributing factor to the results' discrepancy observed among different studies.

In the recent years, growing attention has been drawn to developing methods to cryopreserve primary neurons in a way that preserves the morphology and function as found *in vivo*.^14,20,21^ In fact, several encounters have urged us to examine the possibility of cryopreserving primary neurons and even the intact dissected TG tissues. For example, when a large number of cells are required for experimental executions, a potential low neuron yield might occur due to lengthy and tedious procedures from processing a large quantity of dissected TGs on the day of sacrifice. In the event of unforeseen death of animals, the samples are often wasted due to either insufficient time of preparation and sample processing or limited manpower for isolation. Cryopreservation can therefore be a practical help to overcome these challenges. Our group therefore presents the options to create a high-quality bank of cryopreserved TG neurons by implementing “isolate-and-store” or cryopreserving intact TG tissues by “drop-and-store” methods so that the researchers can have a more flexible planning to obtain a more efficient *in vitro* assays setup.

To the best of our knowledge, there is no report demonstrating the feasibility of using cryopreserved TG tissues or the cryopreserved dissociated TG neurons for studies associated with corneal nerve studies.

In the present study, we aim to establish an optimal setup for isolating primary sensory neurons from mouse TG nerve fibers to address the hurdle brought by commonly adopted isolation method. On this basis, we introduced several fine-tuning adjustments by adding TrypLE treatment to improve tissue dissociation after standard collagenase digestion, followed by putting it through 30/60% discontinuous Percoll (polyvinylpyrrolidone-coated silica) density gradient to achieve efficient isolation with high purity. The isolated TG neurons were maintained in 5-Fluoro-2-deoxyuridine (FUdR) containing media to diminish non-neuronal cells. Furthermore, we also examined the feasibility of cryopreservation by investigating the short- (1 week) and long-term (3 months) cryopreserved effects on the neuronal morphology and neuronal expression. Our work provides a detailed step-by-step guideline for a complete and robust *in vitro* TG culture setup. The positive neuronal expression from the isolated TG neurons, as well as the morphological preservation with distinct neurite features from the cryopreserved TG, further confirmed its great potential of being a standardized model system for corneal nerve studies to provide robust *in vitro* assessments for its future clinical applications.

## Methods

### TG harvesting

**FIG. 1.**
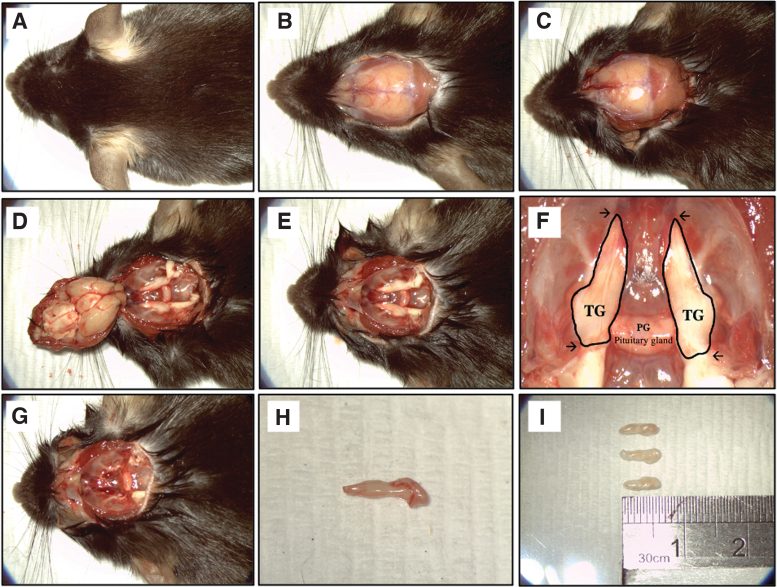
Still photographs of TG collection. **(A)** Apical view of the mice head. **(B)** Midline incision made at the *top* of the head to mid-lumbar region to expose the crown. **(C)** A transverse cut was made at the brainstem to allow the separation of brain from the spinal cord. **(D, E)** Removal of the *top* of the skull and brain to expose the base of the skull and TGs. **(F)** Zoomed in view of the base of the skull. Each mouse contains two TGs that are branched and threaded through the hole on the base of the skull connecting toward the eye. The TG is outlined in *black* with *arrows* indication of the cutting site. **(G)** Apical view after the removal of the TGs. **(H, I)** The collected TG tissues are ∼0.6–0.7 cm in length. TG, trigeminal ganglion.

Mice were euthanized by intraperitoneal injection with an overdosed pentobarbitone sodium (Valabarb^®^). The euthanized mice were placed in a prone position, and a midline incision was made apically using a surgical blade no. 10 from the back of the eyes to the mid-cervical region to expose the crown (Fig. 1A, B), followed by a transverse cut at the brain stem using a Noyes scissor to separate the spinal cord (Fig. 1C). The top of the skull was removed to expose the brain stem and TG (Fig. 1D, E). The symmetric TGs are identified at the base of the skull and are perpendicular to the pituitary gland (Fig. 1E, F). Subsequently, TGs (∼0.6–0.7 cm in length) (Fig. 1G–I) were transferred into Dulbecco's phosphate-buffered saline (DPBS) containing 2% vol/vol Antibiotics–Antimycotics reagent.

### Glass coverslip coating

Autoclaved glass coverslips with 14 mm diameter were used to coat with 50 μg/mL Poly-D-lysine (PDL) solution in DPBS with a volume of 150 μL at room temperature for 1 h. After removal of PDL, the coverslips were washed thrice with sterile water and air dried before another coating with 20 μg/mL laminin in DPBS for 1–2 h at 37°C. The coated coverslips were used immediately or stored in laminin working solution at 4°C and used within 4 days.

### Materials and reagents

Serum-free culture media for culturing mouse primary neurons (B-27 Plus Neuronal Culture System comprising Neurobasal^TM^ Plus Medium and B-27 Plus Supplement) was purchased from Gibco^®^ (Waltham, MA). Other culture reagents required for cell culture were also purchased from Gibco: Collagenase II, Antibiotic-Antimycotic, PDL, Gentamicin, TrypLE™ Select, and N-2 Supplement. Chemicals, including DNase I, Percoll^®^ reagent, FUdR, Dimethyl sulfoxide (DMSO), bovine serum albumin (BSA), and Triton X-100, were from Sigma-Aldrich (St. Louis, MO). Glass coverslips used for cell culture were obtained from Marienfeld-Superior (Lauda-Königshofen, Germany). Pentobarbitone sodium used for euthanatizing the animals was purchased from Valabarb^®^ (Jurox; Rutherford, NSW, Australia).

All the dissecting and sample handling tools scissors and forceps were from Electron Microscopy Sciences (EMS; Hatfield, PA) and were autoclaved before use. Polyclonal anti-beta-III tubulin antibody (Tuj1) and monoclonal anti-SMI312 antibody were purchased from BioLegend (San Diego, CA). The polyclonal anti-vimentin antibody was purchased from Abcam (Cambridge, MA). The anti-NeuN monoclonal antibody was purchased from Millipore (Temecula, CA).

### Isolation of primary sensory neurons from mouse TG

Upon dissection, mouse TG tissues were cut into small pieces (∼1 × 1 mm) and treated with 1 mL of enzyme solution containing collagenase II (5 mg/mL), DNase I (0.2 mg/mL), 2 × antibiotics-antimycotics, and gentamicin (50 ng/mL) in Neurobasal™ Plus Medium in a 1.5 mL microcentrifuge tube. The tissue digestion was kept at 37°C for 60 min with constant agitation at 700 rpm. After trituration, the lysate was centrifuged at 400 *g* for 4 min. The pellet was resuspended in 0.5 mL of TrypLE Select and incubated at 37°C for 3 min before adding 800 μL of Neurobasal Medium Complete (NBM-C; containing 2 × B-27 Plus Supplement, 1 × N-2 Supplement, 1 × antibiotics-antimycotics, and 50 ng/mL gentamicin) and triturate gently for five times using a P-1000 and a P-200 pipette, respectively, to facilitate further dissociation. Finally, the cell lysate was centrifuged at 400 *g* for 4 min, and the pellet was resuspended in 1 mL of NBM-C.

Following enzyme digestions, cell suspension was carefully put through a 70 μm cell strainer in a 50 mL Falcon tube containing 3 mL NBM-C. The filtering step was repeated for another two times to achieve single cell suspension before being carefully loaded above the 30/60% Percoll gradient solution in a 15 mL tube with 4 mL of each gradient prepared with NBM-C. Subsequently, the neuronal fraction of the sample was obtained after centrifugation at 1200 *g* for 10 min. To illustrate further, the non-neuronal fraction was observed at the interphase between 0% and 30% gradient layer (Supplementary Fig. S1) which was discarded. The neuronal fraction accumulated at the 30% gradient layer and the interphase between 30% and 60% Percoll layer (Supplementary Fig. S1) was collected using a 1000-micropipette in a 50 mL Falcon tube followed by adding NBM-C with at least sixfold of volume for Percoll solution removal. It is worth mentioning that the pipette tips used to collect the non-neuronal fraction were discarded, and fresh pipette tips were used to collect the 30/60% interphase layer to minimize the contamination of non-neuronal cells.

Finally, the sensory neuronal cells were then pelleted after centrifugation at 670 *g* for 4 min and were further resuspended for cell seeding or cryopreservation. The neuronal culture was maintained in NBM-C containing 20 μM of FUdR to prevent overrun of non-neuronal glial cells, and the medium was changed every other day.

### Cryopreservation method

Intact TG tissues and isolated primary neuron cells were cryopreserved in NBM-C medium containing 45% fetal bovine serum (FBS) and 10% DMSO as previously described.^14^ The effects of cryopreservation were further evaluated after short-term (1 week) and long-term (3 months) cryo-storage by comparing the morphology and neuronal expression with the freshly prepared culture.

## Experiment

### Experimental design

#### Animals

A total of 20 C57BL6 male mice (Jackson Laboratory, Bar Harbor, Maine) were used in this study. All animal housing was conducted under the guidelines of Association for Research in Vision and Ophthalmic Statement for the Use of Animals in Ophthalmic and Vision Research. The protocol was approved by the Institutional Animal Care and Use Committee of SingHealth, Singapore (IACUC Reference Number: 2020/SHS/1612).

#### *In vitro* TG neuron culture

**FIG. 2.**
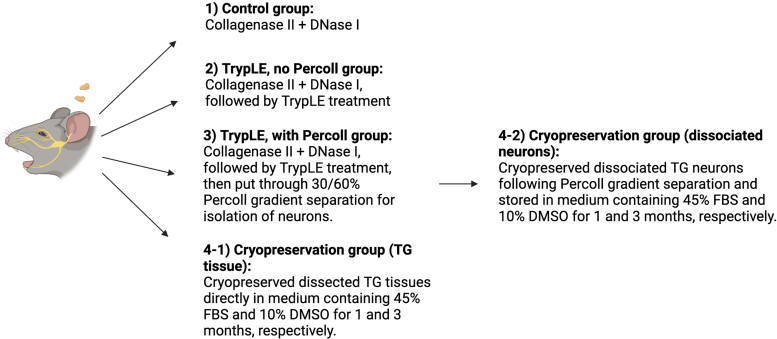
Descriptive image of the experimental design used in the *in vitro* TG neuron culture (Created with BioRender.com).

After euthanization, the mice TG was harvested according to the description in the Methods section. Following the TG collection, the TGs were randomly divided into four groups to examine the isolation efficiency of the TG culture: (1) Control group (Isolation of TG neurons using only collagenase II and DNase I, (2) TrypLE, no Percoll group (Additional dissociation step using TrypLE Select following collagenase II and DNase I treatment), (3) TrypLE, with Percoll group (Further purification step using 30/60% Percoll gradient separation). (4) Cryopreservation group (TG tissues or isolated TG neurons using optimized method) (Fig. 2). We hypothesized that the TrypLE with Percoll group will generate high yield with homogenous primary neuronal culture with high reproducibility, and this optimized method can be used as a standardized model system for regenerative corneal nerve studies. Using the optimized method, we have further accessed the feasibility of cryopreservation of TG tissue and dissociated TG neurons.

#### Immunocytochemical staining

For investigation of the cell morphology and evaluation of the neuronal expression of the primary neuronal culture, cells were fixed with 4% paraformaldehyde for 30 min at room temperature, blocked, and permeabilized in blocking buffer comprising DPBS supplemented with 5% normal goat serum, 2% BSA, and 0.1% Triton X-100 before incubating in respective primary antibodies of the neuronal/glial markers (Supplementary Table S1): Class III β-Tubulin (Tuj1, neuron specific marker), neuronal nuclear protein (NeuN, neuron soma specific marker), and SMI312 (Axon specific marker).^22^ Glial cell-specific vimentin antibody^23^ was also double-stained to validate the purity of neuron culture.

#### Neurite outgrowth quantification

To validate the optimized isolation protocol established in this study, neurite outgrowth observed following the immunocytochemical staining was further analyzed (number of neuronal bodies, total neurite length, number of neurite nodes, and number of neurites) using the NeuronJ plugin in Fiji/ImageJ software.^24^ In parallel, same neurite outgrowth analysis was also performed on the cryopreserved group to examine its potential of being used as cell sources for the *in vitro* TG culture model. All data were expressed as mean ± standard deviation (*n* = 3 images/group). The statistical analysis was carried out by Kruskal–Wallis test using STATA (STATACorp., College Station, TX) to compare the differences across all groups, and a *p* < 0.05 was considered as significant.

## Experimental Results

### Cell morphology

**FIG. 3.**
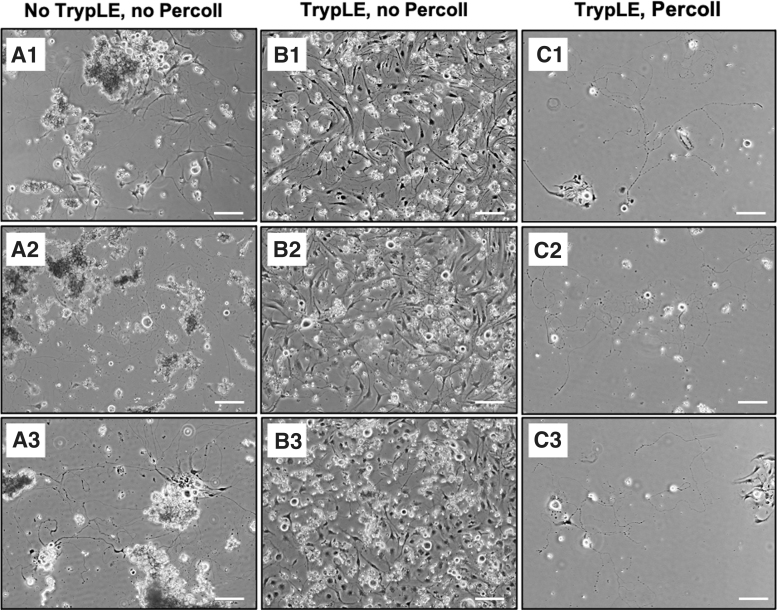
Bright field microscopic images of commonly adopted isolation method of primary sensory neurons from mouse TG as experimental control group. (**A1–3**) TG culture obtained using enzymatic digestion without TrypLE Select treatment results in attachment of undigested tissue pieces and cell debris. (**B1–3**) Additional treatment using TrypLE Select at 37°C helped to dissociate the undigested tissue pieces to generate higher neuron yield. (**C1–3**) Tissues further treated with TrypLE Select followed by Percoll gradient separation greatly reduced the density of non-neuronal fraction in the culture. Triplicate images per isolation method are taken at day 3 by Nikon inverted microscope (Nikon Eclipse TS100, Japan; Magnification: 10 × ; Scale bar = 100 μm).

The cell morphology of the isolated primary neuron cells can be identified using phase-contrast microscopy. The mouse TG culture contains heterogenous cell populations, which include neuron and non-neuronal cell types. Obtaining single cell suspension with minimal interference from non-neuronal cell types for *in vitro* TG neuron culture is essential for downstream characterizations and application for corneal nerve studies. The commonly adopted isolation method of using only collagenase digestions^25–28^ was repeated in this study as control group and has resulted in several undigested tissue pieces, cell debris, and clumps of neuron cell bodies as observed (Fig. 3A). The presence of these resulting tissue and cellular remnant could limit the visualization of the source of neurite outgrowth, thereby posing challenges for its downstream characterization and quantification analysis. Besides, it could also hinder the cell counting procedure and subsequently affect the accuracy and reliability when applying this model for potential drug testing in the *in vitro* setting.

In contrast, additional TrypLE Select treatment following collagenase digestions enabled further cell dissociations, reducing cell debris and cell clumping (Fig. 3B). However, non-neuronal cells seen in Figure 3B have overtaken the TG culture; the overlay between non-neuronal cells and neuronal cells makes the soma undistinguishable for neurite outgrowth observation. Such challenges were overcome by implementing additional step of 30/60% Percoll gradient separation, effectively reducing the presence of non-neuronal fractions (Fig. 3C).

### Neurite outgrowth from TG neurons

**FIG. 4.**
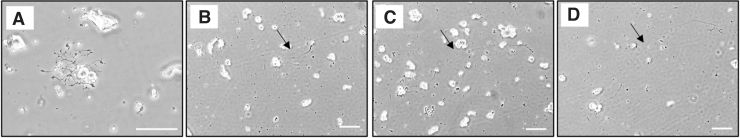
Mouse TG neurons culture. Light microscopic images of neurite outgrowth observed at **(A)** 2 h, **(B)** 2, **(C)** 3, and **(D)** 5 days after cell seeding on PDL and laminin double coated glass coverslips. The neurite was lengthened (*black arrow*) over the course of culture. Images are taken by Nikon inverted microscope (Nikon Eclipse TS100, Japan; Magnification: (**A**) 20 × , (**B–D**) 10 × ; Scale bar = 100 μm). PDL, Poly-D-lysine.

Using the optimized isolation protocol, the outgrowth of isolated neurites from soma was observed under bright-field using an inverted microscopy with phase-contrast objectives at 2 h (Fig. 4A) after plating the dissociated TG neurons onto the coated coverslips and was extended during 5 days of *in vitro* culture (Fig. 4B–D). These observations confirm the capacity of neuronal growth in the *in vitro* culture setting.

### Neuronal morphology of cryopreserved TG tissues and dissociated TG neurons

**FIG. 5.**
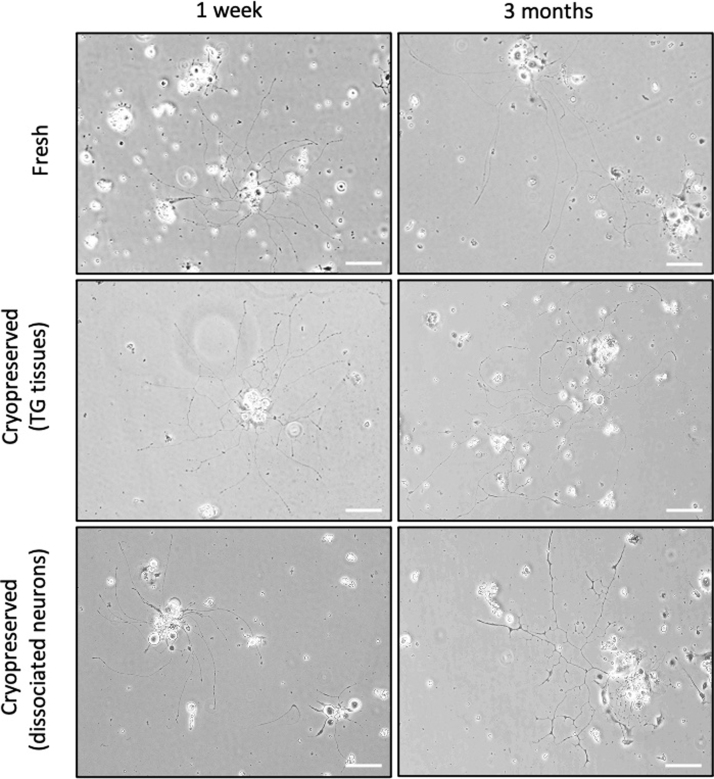
Representative bright field microscopic images of *in vitro* cultured mouse TG neurons from freshly prepared, cryopreserved TG tissues and cryopreserved dissociated neurons for 1 week and 3 months, respectively. Images were taken at day 3 by Nikon inverted microscope (Nikon Eclipse TS100, Japan; Magnification: 10 × ; Scale bar = 100 μm).

In comparison to the freshly prepared culture, we observed that the neurite outgrowth and morphology examined using the same inverted microscopy were comparable in the cryopreservation groups at post 1 week and post 3 months' time (Fig. 5).

### Protein expression

**FIG. 6.**
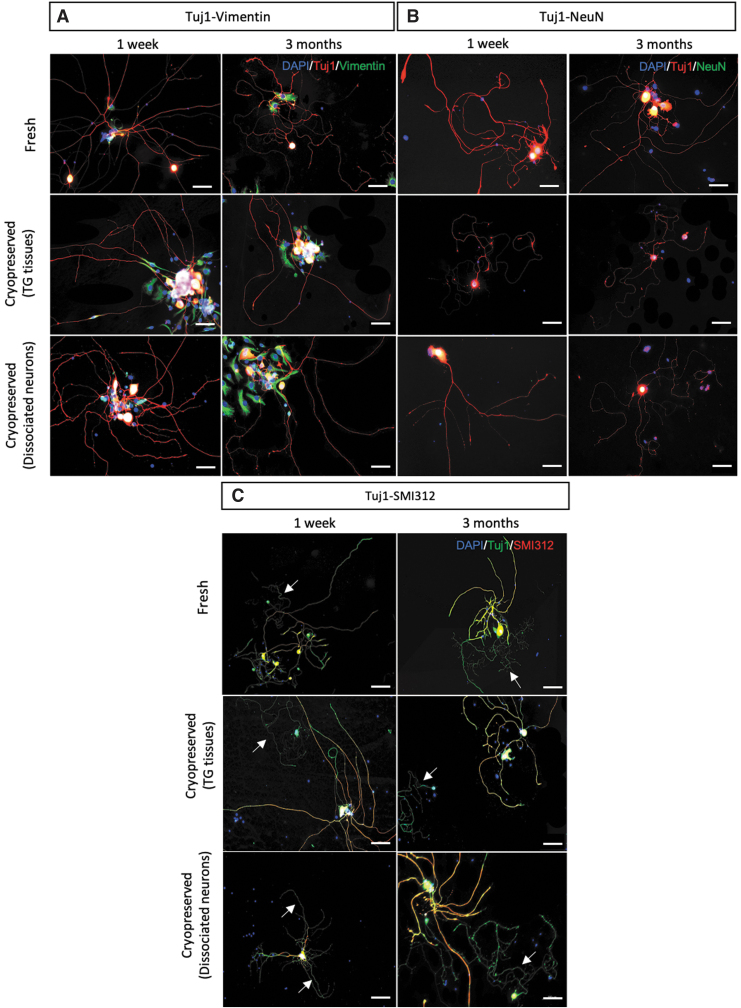
Representative fluorescence microscopic images of *in vitro* cultured TG neurons. Freshly cultured and cryopreserved TG tissues and dissociated TG neurons were double stained with **(A)** neuron specific Tuj1 (*red*) and pan-glial vimentin (*green*) markers (Magnification: 20 × ; Scale bar = 50 μm). **(B)** Tuj1 (*red*)-NeuN (*green*) double staining was also performed to discern the soma localization (*yellow*) of the neuronal cells, while the non-neuronal cells stained only with Hoechst nuclear stain in *blue* (Magnification: 20 × ; Scale bar = 50 μm) **(C)** As SMI312 (*red*) is pan-axonal markers, the Tuj1-positive neurites (*green*) with SMI312-negative expression indicate the presence of dendrites (*white arrow*) while the axons are costained with anti-Tuj1 and anti-SMI312 antibodies (*yellow*; Magnification: 10 × ; Scale bar = 100 μm). The Nuclei were stained with Hoechst dye (*blue*). The cultured TG neurons were fixed at day 3, and the fluorescence images were taken by a fluorescence microscope (Carl Zeiss, Oberkochen, Germany).

The specific neuronal expression can be validated with immunofluorescence staining using a fluorescent microscope. The neurite features should express the neuron specific Tuj1 marker and should be absent in expressing glial cell markers such as vimentin staining. Figure 6A shows the expected results of neurites expressing Tuj1-positive staining without colocalizing with vimentin-positive staining, confirming the successful isolation of TG neuron cells. The positive vimentin staining on the neuronal nuclei was associated with its intrinsic properties of nuclear envelope attachment.^29^ The soma-specific marker, neuronal nuclear protein (NeuN), also further substantiated the localization of the neuronal cells (Fig. 6B).

Of note, the Tuj1-SMI312 double staining results in which Tuj1 stained both dendrites and axons, the pan-axonal marker SMI312 staining distinguished the expression of axons from dendrites (Fig. 6C). There was no apparent difference in the length of neurite outgrowth in all groups, although slightly thinner neurites at times were noticed in the culture seeded with cryopreserved dissociated neurons thawed after 3 months storage when in comparison to the culture prepared from cryopreserved TG tissues (Fig. 6B). In addition, the density of neurite outgrowth in the cryopreserved TG tissue groups appeared to be comparable to the freshly seeded group, while reduced quantity of neurite outgrowth was observed in the cryopreserved dissociated neuron groups. Nevertheless, the comparable length and morphology of typical long thread-like axon and short branched dendrite were found in all groups regardless of the cryopreservation status and duration, indicating the potential application of DMSO-based cryopreserved TG tissues or cryopreserved dissociated neurons as *in vitro* model for corneal nerve studies.

### Neurite outgrowth analysis

**FIG. 7.**
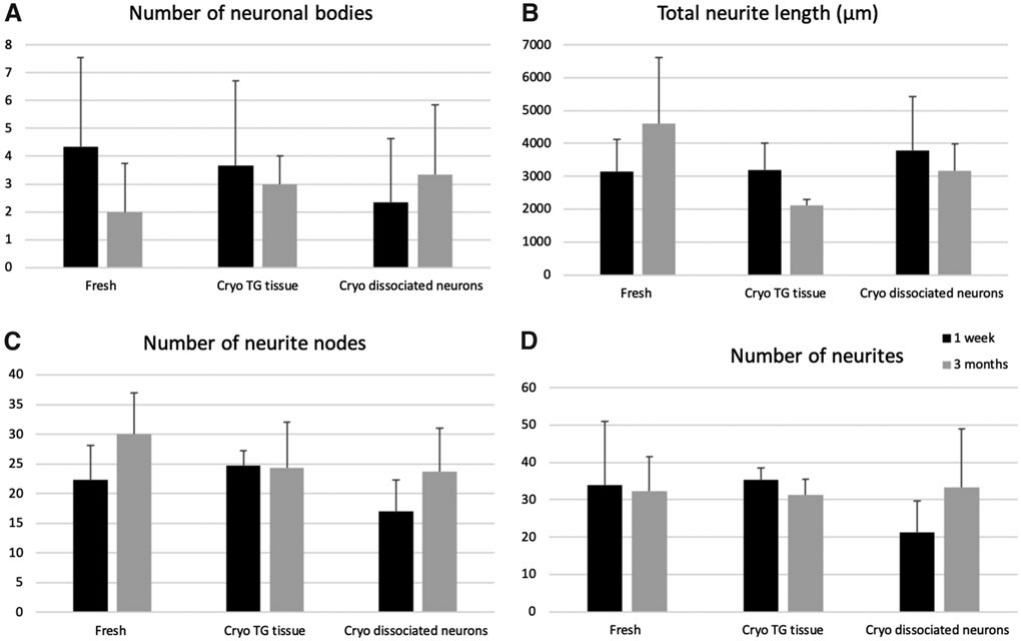
Quantitative assessments of neurite outgrowth from freshly isolated culture and culture prepared with cryopreserved samples. Following the immunocytochemical staining, neurite outgrowth parameters: **(A)** number of neuronal bodies, **(B)** total neurite length, **(C)** number of neurite nodes, and **(D)** number of neurites were analyzed using the NeuronJ plugin in Fiji/ImageJ software. Data represent mean ± SD (*n* = 3 images/group). SD, standard deviation.

The validity of the optimized protocol can be assessed by performing quantification analysis on the neurite outgrowth from the isolated neuronal culture. Figure 7 displays the quantitative assessments of neurite outgrowth from the optimized isolation protocol (neurons were freshly isolated) and from the culture prepared using either cryopreserved TG tissues or dissociated neurons after short-term (1 week) and long-term (3 months) cryo-storage. All four examining neurite parameters, number of neuronal bodies (Fig. 7A), total neurite length (Fig. 7B), number of neurite nodes (Fig. 7C), and number of neurites (Fig. 7D) showed comparable results across all three groups. Although lesser neurites (21.33 ± 8.33; *p* = 0.17) and neurite nodes (17.00 ± 5.29; *p* = 0.58) were traced in the culture of cryopreserved dissociated 1 week group (Fig. 7C, D) when in comparison to freshly prepared group (number of neurites: 34.00 ± 17.1; number of neurite nodes: 22.33 ± 5.77), the differences were not significant.

Our quantitative results further confirm the qualitative results observed (Fig. 6), substantiating the optimized isolation protocol established in this study, as well as the potential use of cryopreserved samples as promising cell sources for corneal nerve studies.

## Discussion

Sensory neuron culture is a representative model as it has the capacity to reproduce the diverse neuronal cell types and response properties seen *in vivo.*^15^ In ophthalmology, sensory neurons are often grown *in vitro* and cocultured with corneal epithelial cells to examine their role in corneal wound healing or reinnervation potential and its underlying mechanisms.^30–32^ Given the known similarity of mature dorsal root ganglion and TG transcript expression profiles, both are popular models used for studying corneal nerve regeneration potential.^33,34^ From an anatomical, as well as a physiological standpoint of corneal nerves being the extension of the TG sensory nerve fibers, a robust approach in isolating TG neuron with the capacity to establish it within an *in vitro* culture is invaluable as a representative model for studies associated to corneal nerves. In this regard, recent studies have adopted the isolated sensory neurons as model system to study neurotrophic effects on neurite outgrowth,^25^ growth factor-dependent neuronal survival,^35^ and potential drug testing^26^ to further contribute the development of therapeutic strategies.

Among the several TG neuron isolation methods summarized in Table 1, performing enzymatic digestions alone with simple mechanical trituration is the most commonly adopted method due to its simplicity.^25–28,36^ However, in the recent years, there has been a growing interest in introducing Percoll gradient based approach to purify neuronal cells^15,37–40^ due to low neuron yield associated with insufficient dissociation or high ratio of non-neuronal cells associated to over-digestion. Indeed, from our thorough evaluations of individual methods, we have also observed the undigested big TG tissue pieces with floating debris (Fig. 3A), and when TrypLE Select treatment was in place to assist in further dissociation, the culture was overrun by the high fraction of non-neuronal cells (Fig. 3B). We have addressed this potential hurdle by reducing the TrypLE Select treatment duration, as well as putting enzymatic treated single cell suspension through discontinued Percoll gradient. However, the challenges remain when Percoll gradients among studies vary.^15,37–40^

**Table 1.** Commonly Adopted Isolation Methods of Primary Sensory Neurons from Murine Trigeminal Ganglion

Isolation method	Cell origin	Principles	Advantages	Limitations	Ref.
Enzymatic digestion	Sprague-Dawley rats (E15) or mouse TG	Specific enzymatic activity can be activated under its optimal working temperature, assisting the release of contained cells from big tissue pieces.	The most common method for primary cell isolation.	The optimal concentration and duration of enzymatic digestions need to be determined to ensure proper tissue digestions with little or no big cellular fragment leftovers which could impede the downstream assessments.	^25–28^
Mechanical dissociation	Sprague-Dawley rats (E15) or mouse TG	By exerting mechanical forces to physically dissociate the cell/tissue clumps through various sizes of glass Pasteur pipettes.	Easy and affordable method. Users can self-determine the orifices size of the fire-polished glass Pasteur pipettes.	Fabricating specific orifices size of fire-polished glass Pasteur pipette could be time consuming and challenging for beginners. Strict safety regulation might limit the implementation. Inadequate mechanical dissociation could impose strain on cells ^45^ and potentially lead to declined cell viability.	^25–28^
Percoll density gradient	Mouse TG	Based on the principle of isopycnic separation, varying densities of cells within a heterogeneous sample will sediment to its isopycnic point upon centrifugation.	Higher neuron vs. non-neuron yield, compensating the limitation of tissue trunk results from improper enzymatic dissociation.	A variety of Percoll gradients were reported from different authors. Therefore, each user might need to perform preliminary optimization to achieve adequate balance between neuronal and non-neuronal fraction.	^15,37–40^
FACS	Mouse TG/DRG (Sensory neurons were labelled in Avil-GFP transgenic mouse)	Based on fluorescence-assisted cell sorting technique.	High purity.	Cell clumps results from insufficient enzymatic digestions could pose threats to the sorting procedures. This method involves in transgenic model and would require addition of multiple growth factors to support the neuronal culture.	^33^

Avil: advillin; DRG, dorsal root ganglion; FACS, fluorescence activated cell sorting; GFP, green fluorescent protein; TG, trigeminal ganglion.

In this context, we have also resolved the challenges by further investigating three commonly used Percoll gradient combinations: 12.5/28%, 20/50%, and 30/60% and examining each layer carefully (only 30/60% data shown in Supplementary Fig. S1). We found that neuronal yield was comparable in 20/50% and 30/60%, while 12.5/28% had the lowest yield (data not shown). It is worth noting the presence of small portion of non-neuronal cells even through purification step by Percoll gradient separation. However, it is reported that an adequate presence of glial cells is essential for supporting and neuronal growth *in vivo*.^41^

Collectively, we have established the optimized *in vitro* culture setup with refinement of culturing parameters. The combination of enzymatic digestion, inclusive of quick TrypLE Select treatment, mechanical trituration, and Percoll gradient separation has greatly improved the ratio of dissociated neurons and non-neuronal cells. Culturing the isolated neurons on the laminin and PDL coated coverslip promotes neurite outgrowth and neuronal cell adhesion, respectively.^42–44^ This method was tested with good repeatability and reproducibility and could therefore be a standardized method for isolating primary sensory neurons from mouse TG for potential studies involved in corneal nerve or corneal neuropathy. Cryopreserving the TG tissues and dissociated neurons with preserved capacity of neuronal growth offers large benefits in terms of flexibility in experimental design, increase reproducibility, and facilitate collaboration among laboratories.^14^

In fact, the resources for the *in vitro* culture setup can be organized in advance to reduce the potential wastage. For example, cryopreserving dissociated TG neurons would enable users to better keep track on the number of neurons obtained per isolation, allowing more accurate estimation of coverslip coating for the subsequent experimental planning. Besides, when a certain number of dissociated neurons are expected in experimental designs, pooling of cryopreserved dissociated neurons could be an advantage of overcoming the potential low yield of neurons upon lengthy and tedious isolation. In contrast, cryopreserving TG tissues immediately after TG dissection could also be a convenient option for researchers to consider. With this option, the sample processing procedures no longer need to strictly abide to the schedule of animal scarification. Besides, this simple “drop-and-store” method creates more rooms for planning a large batch of animal sacrifice for subsequent experiment executions or when cutting down the cost of animal maintenance fee is necessary. In addition, in the event of unforeseen death of animals, a quick procedure of cryopreserving TG tissues can salvage the potential loss of samples due to unprepared *in vitro* setup such as coverslip coating.

While DMSO is a commonly used cryoprotectant for freezing cells,^45,46^ commercially available DMSO-free cryoprotectants are also of great interest for clinical applications such as cell-based therapy due to its high viability and low cytotoxic functions. In fact, studies have demonstrated high post-thaw cell viability in cells cryopreserved using non-DMSO cryoprotectant compared with DMSO-cryopreserving cells.^47,48^ In this study, we have cryopreserved the dissociated TG neurons using both DMSO and DMSO-free cryoprotectants (CryoScarless), respectively, using our optimized protocol described above. However, no cells were found attached in the CryoScarless preserved group (data not shown), suggesting that CryoScarless might not be suitable in the cryopreservation of neuronal cells. The present study showed the preservation of TG neuron cell viability and morphologies after being cryopreserving in media containing 45% FBS and 10% DMSO for up to 3 months, thereby confirming DMSO being a suitable cryoprotectant. This is further substantiated by the positive expression of respective neuronal markers from immunocytochemical staining of each experimental group.

Previously, Liu and colleagues have investigated long-term (1 month) cryo-storage effect of corneal lenticules.^49^ In this study, we chose 3 months as the long-term cryopreservation evaluation time point as it provides adequate timeframe for researchers to plan the associated experiments without delaying the project progress. As expected, our observed results are in consensus with a similar study investigating the long-term cryopreservation effects with over 1 year storage on primary hippocampal and cortical neurons and human fetal neuronal cells,^14,50^ suggesting that the successful maintenance of neural morphology is feasible. From these studies, the preservation of cellular and functional characteristics such as ultrastructural integrity, development, and formation of functional synapses was also reported. And most significantly, the capacity in response to extracellular stimulation remains unchanged.^14,50,51^ These findings support the tremendous potential of cryopreserved TG tissues and dissociated TG neurons, which require further validation over a longer cryopreservation timeframe with more cellular and functional analysis.

In this study, two possible options of mouse TG cryopreservation were demonstrated—(1) the dissociated TG neurons and (2) the intact TG tissues upon TG dissection. The subsequent morphological and immunocytochemical evaluations showed equivalent patterns compared with freshly plated TG neurons. During the course of culture, the length and the morphology of neurite outgrowth remain comparable in all groups (Figs. 6 and 7B), while slightly thinner and less neurites were observed at times, only in the dissociated neuron group after cryopreservation, but not in the whole TG tissue cryopreservation group (Figs. 6 and 7D).

The possible explanation for the reduced quantity and thinner neurite outgrowth noticed in the group seeded with cryopreserved dissociated neurons could be due to the fact that the dissociated neurons have higher susceptibility to cryopreservation procedures, while the undissociated neurons were better ensheathed by the outer collagenous layer of intact TG tissues.^52^ Still, the rest of the morphological characteristics such as length and distinct neurite morphology observed in both cryopreserved groups remain similar to freshly isolated group using optimized method (*p* > 0.05; Figs. 5–7B) and could be useful *in vitro* models for further studies.

Our *in vitro* culture setup has demonstrated the preservation of neuronal growth capacity, as well as its morphology, and that DMSO is a useful cryoprotectant for this model. We have also validated the reproducibility by inter-researchers' performance. The obtained consistent neuronal cultures further confirm the potential of using it as a standardized neuronal cell model *in vitro* and is well-suited for performing assessments such as neuronal therapeutics or stimuli responsiveness, safety, and toxicity when considering to evaluate potential treatment for corneal reinnervation.^14,15^

The present protocol is a pivotal pilot study for *in vitro* corneal nerve studies. However, limitations of our studies include the lack of more refined assessments such as biochemical signaling and electrophysiological studies using patch clamping and chloride imaging, which may be used to evaluate the effect of cryopreservation over the functional quality of culture^15,28^ to further validate the *in vitro* model. The concerns regarding possible endotoxin contamination brought by Percoll have been discussed,^53,54^ and might have contributed to the reduced number of neurites and neurite nodes observed in the culture prepared from cryopreserved dissociated neurons. Further investigations on the cytotoxicity effect from Percoll and density-matching reagents such as OptiPrep™ are therefore warranted in the next step of the study for further improvement. Future direction may also include the development of 3D *in vitro* culture to better study the corneal nerve under physiologically relevant culture conditions.

## Conclusion

In this article, we have described the refined step-by-step procedures to dissect, isolate, and culture primary sensory neurons from mouse TG. We have highlighted the adjustable factors that could greatly improve the purity of heterogenous TG neuron culture and further validated it by examining specific neuronal markers with immunofluorescent staining. In addition, we have demonstrated the minimal effect of cryopreservation agents by showing the morphological preservation of TG neurons in cryopreserved TG tissues and dissociated TG neurons compared to a control group cultured with freshly prepared TG neurons. With this protocol, we have successfully improved the *in vitro* setup with consistent neuron cultures. Our work not only suggests the plausibility of this refined *in vitro* setup being a standardized model system for corneal nerve studies, the great benefits of using cryopreserved TGs also highlight its potential of being a promising cell source for evaluating potential treatment for corneal reinnervation.

## Supplementary Material

Supplemental data

Supplemental data
