# Supplementary material for: Culture of Primary Neurons from Dissociated and Cryopreserved Mouse Trigeminal Ganglion
Source: Tissue Eng Part C Methods. 2023 Aug 8;29(8):381–93. doi: 10.1089/ten.tec.2023.0054 (PMC10442681; doi:10.1089/ten.tec.2023.0054)

**Figure Legend (Supplementary Material)**

FIG. S1. Primary sensory neuronal fraction isolation with Percoll gradient by collecting neuronal fraction at 30% and the interphase between 30 and 60% Percoll. (**A**) The filtered single cell suspension was laid over 30% and 60% of Percoll gradient layer with 4 ml each prepared with NBM-C before Percoll gradient separation, and (**B**) after centrifugation at 1,200 ×g for 10 minutes. (**C**) Each layer (0%, 0/30% interphase, 30%, 30/60% interphase, 60% and “pellet”) was collected and seeded on PDL- and laminin-coated coverslip (one coverslip per layer) to examine the distributions of neuronal and non-neuronal fraction at each layer at day 1 (C1, 3, 5, 7, 9, 11) and day 3 (C2, 4, 6, 8, 10, 12), respectively. After three days culture in culture media, neurite outgrowth was observed from neuron cell bodies (blackhead arrow) under Nikon inverted microscope (Nikon Eclipse TS100, Japan; Magnification:10×; Scale bar = 100 μm).


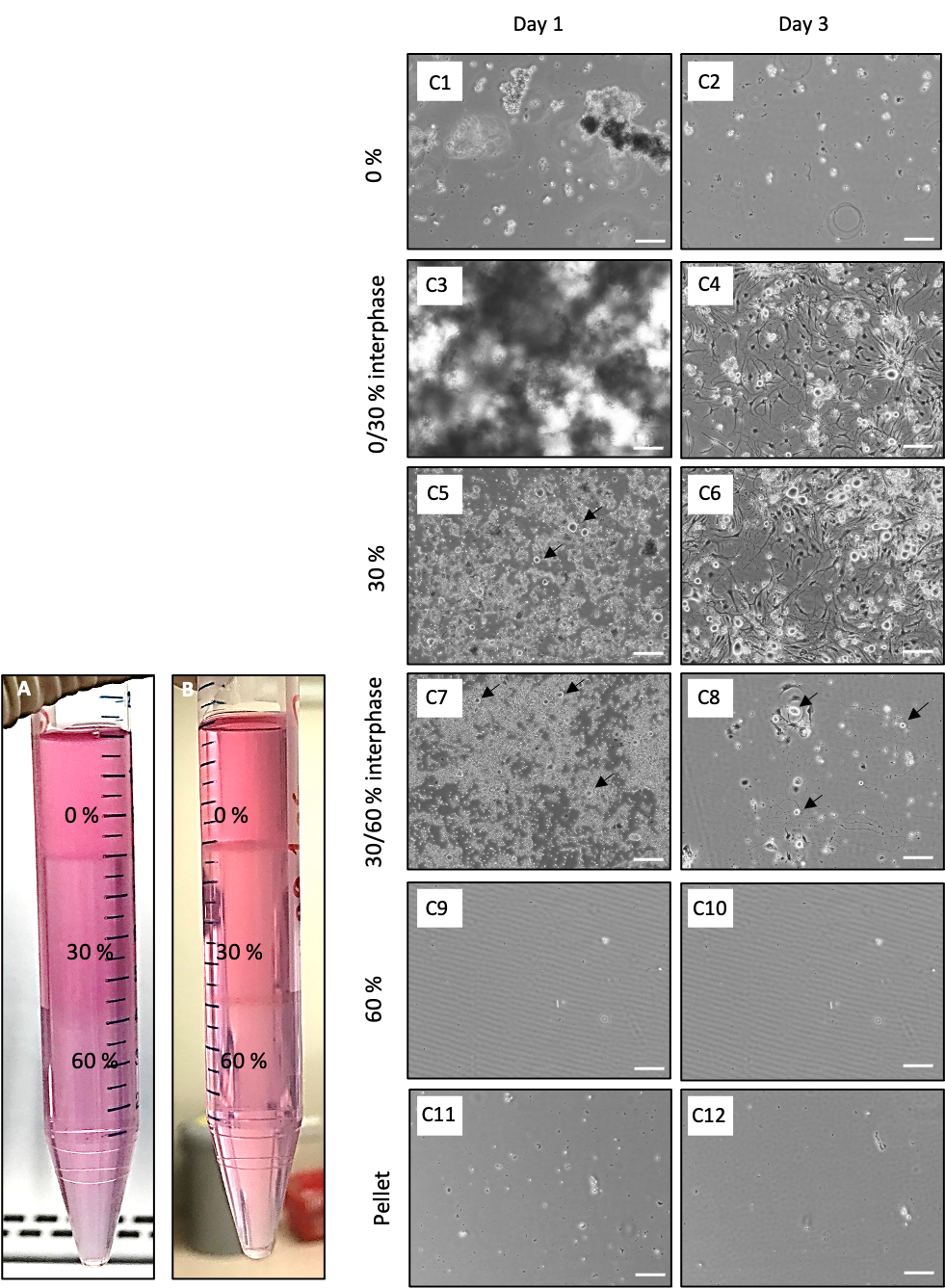

Supplement: Supplemental data [file Suppl_FigS1.docx]
