# Supplementary material for: Culture of Primary Neurons from Dissociated and Cryopreserved Mouse Trigeminal Ganglion
Source: Tissue Eng Part C Methods. 2023 Aug 8;29(8):381–93. doi: 10.1089/ten.tec.2023.0054 (PMC10442681; doi:10.1089/ten.tec.2023.0054)
Supplement: Supplemental data [file Suppl_TableS1.docx]

**Supplementary Materials**

Table S1. Primary antibody source and dilution.

| **Antibody Target** | **Source** | **RRID** | **Dilution** |
| --- | --- | --- | --- |
| Beta-III tubulin (Tuj1) | Rb polyclonal, Biolegend (802001) | AB_2564645 | 1:150 |
| Vimentin | Ck polyclonal, Abcam (ab24525) | AB_778824 | 1:150 |
| NeuN | Ms monoclonal, Millipore (MAB377) | AB_2298772 | 1:100 |
| SMI312 | Ms monoclonal, Biolegend (837904) | AB_256678 | 1:100 |
